# Precision Medicine and Exercise Therapy in Duchenne Muscular Dystrophy

**DOI:** 10.3390/sports7030064

**Published:** 2019-03-15

**Authors:** Matthew Kostek

**Affiliations:** 1 Laboratory of Muscle and Translational Therapeutics, Department of Physical Therapy, Duquesne University, Pittsburgh, PA 15228, USA; kostekm@duq.edu; Tel.: +1-412-396-5546; 2 McGowan Institute of Regenerative Medicine, University of Pittsburgh School of Medicine, Pittsburgh, PA 15228, USA

**Keywords:** exercise, dystrophy, SNP, muscle contraction, biomarkers, gene therapy, precision medicine, precision exercise, therapy

## Abstract

Precision medicine is being discussed and incorporated at all levels of health care and disease prevention, management, and treatment. Key components include new taxonomies of disease classification, the measurement and incorporation of genetics and “omics” data, biomarkers, and health care professionals who can optimize this information for a precision approach to treatment. The study and treatment of Duchenne Muscular Dystrophy is making rapid advances in these areas in addition to rapid advances in new gene and cell-based therapies. New therapies will increase the variability in disease severity, furthering a need for a precision-based approach. An area of therapy that is rarely considered in this approach is how the physiology of muscle contractions will interact with these therapies and a precision approach. As muscle pathology improves, physical activity levels will increase, which will likely be very beneficial to some patients but likely not to all. Physical activity is likely to synergistically improve these therapies and can be used to enhance muscle health and quality of life after these therapies are delivered using the tools of precision medicine.

## 1. Introduction

The idea of personalized medicine has been gaining significant interest since the sequencing of the human genome. Treatment for most of the major chronic diseases and many disciplines within medicine having been attempting to adopt or at least are considering how to adopt the idea [1,2,3]. Scientists and clinicians are even discussing the idea of how “exercise” can be utilized and incorporated into a personalized medicine approach [4]. With a slightly modified and updated approach, the National Research Council used the term “precision” medicine instead of personalized medicine [5]. Precision medicine, they suggest, relies on the ability to subcategorize individuals within a disease into those who will respond to specific targeted therapies and those who would not respond, or for whom the treatment might even be detrimental. This will require, they explain, new taxonomies of disease where much more information is brought to bear for each patient, including ‘omics’, lifestyle and diet, biomarkers, fully digitized clinical histories, and measures that still may be in development. New and encompassing analyses will collectively utilize and incorporate this data into a precise treatment. Ideally, biomarkers would then allow precise tracking of the effectiveness of the treatment. The concept and practice of personalized medicine is a lofty goal and not without its share of valid criticisms, particularly, the aspects related to genetics (e.g., SNPs) as disease modifiers [6,7,8]. This problem is acknowledged, yet DNA sequence variation may only play a small role or eventually be superseded by other omic data; this type of biologic big data is only one concept of precision medicine, and the possible failure of that one concept does not doom the model or precision medicine, itself. It has even been suggested to view personalized medicine as dependent upon genetics, even though precision medicine relies heavily on data, analytics, and information [9]. Recognizing these definitions, the remainder of this report will focus on precision medicine. Recent advancements in each of these concept areas of precision medicine, and many new treatments, are being reported for the genetic disease Duchenne Muscular Dystrophy (DMD). A clear path in the field of DMD, however, has not yet been mapped. Precision medicine will be more challenging than common chronic diseases, as sample sizes in DMD studies are relatively small, and not nearly as much is known in terms of biomarkers or additional genetic variation, such as SNPs. The many new treatments also will undoubtedly modify (and inform) a precision medicine approach [10]. Progress is being made in these areas, but an area that is lacking is discussion of the use of therapeutic exercise as part of a precision medicine approach. As treatments for DMD improve, muscle function and physical activity will inevitably increase. How will the increased physical activity or exercise rehabilitation accompany and interact with these new treatments? To ignore the importance of the physiologic effect of increased muscle contractions (i.e., physical activity) in this disease is missing important opportunities to not only improve these treatments but to be ready to optimize the entire intervention program. In this perspective paper, the basic concepts of precision medicine will be examined in relation to DMD, with a special emphasis on how therapeutic exercise programs might interact with each of these concepts. The goal is to highlight the likely interactions of precision DMD medicine with therapeutic exercise, and how this should be considered an integral part of these treatments.

## 2. Variance in DMD Disease Progression

Because there is no cure for DMD, and boys with DMD are typically dependent upon a wheelchair before the age of 20, with a life expectancy of less than 30 years of age, it can be easily underappreciated how variable the disease is. The natural history of disease varies substantially amongst patients with DMD. While the age of death has increased from less than 20 years to the late 20s [11,12], some patients survive into their 40s [13]. These long-lived patients are not receiving the newest advanced treatments of gene therapy or exon skipping but instead demonstrate disease variability. This extended life span has occurred through improved and optimized treatment, improved corticosteroid prescription, and more comprehensive cardiac and pulmonary treatments. The cardiac and pulmonary care is particularly noteworthy, because failure of these organs is the primary cause of mortality. For DMD precision medicine, these organs pose a special concern because they are sometimes not affected by the most advanced treatments, even though skeletal muscle is affected. Exercise also poses specific demands on the heart and diaphragm in comparison to skeletal muscle. Thus, cardiopulmonary concerns warrant special attention somewhat beyond the scope of this review. Because of this importance, they will be referenced throughout, but the primary focus here will be skeletal muscle. There are not enough data available in most instances to make valid conclusions about the cardiopulmonary system in this regard. Much research and special attention is needed in this area. As optimized treatments continue to extend lifespans, they also uncover the variability and other underlying differences, such as genetic modifiers, of disease. Even within rare families with siblings who have DMD, there can be substantial variability in disease severity, such as cardiac and pulmonary functions [14]. One of the largest databases and studies to date, UMD-DMD France, contains 2411 entries of patients with DMD and complete data on 278 patients, with an average of a 14 year follow up for each patient. In an analysis of this database, patients were statistically classified into three distinct groups based on disease severity. Class A was the most severe, class B was moderate, and class C was the least severe [15]. Loss of ambulation measures are often considered the most complete measure of physical function and most reliable measurements of DMD disease progression. In this analysis, one ambulatory measure, “never acquiring running ability”, affected 43% of patients in the most severe group A, with only 18% and 8% in the less severe groups, B and C. Several additional measures confirmed this severity grouping, reinforcing the idea of variability in physical function. Within this database itself the disease has even been classified as both DMD and Intermediary Muscular Dystrophy (IMD) because of the variability in disease progression [15]. The cause of this extensive variability did not appear to be attributable to treatment or any obviously identifiable cause. The variability in disease progression and severity at any one time point, for example, showed essentially no association with dystrophin mutation. However, there were differences in dystrophin expression in biopsies, with the severe group A showing no dystrophin in any fibers, while the moderate group had 13% of fibers and the least severe group C showed 18% of fibers expressing dystrophin protein in immunohistochemical imaging. Nevertheless, only one patient had a detectable protein level on western blot. Thus, while some revertant fibers existed in the least severe grouping it was not enough to be detectable on western blots. The cause of revertant fibers is unknown, but it is likely that patients with an increased number of revertant fibers had less severe disease. The revertant fibers may then explain some of the variability. Variance in disease severity is at least partially controlled by other genetic differences beyond dystrophin. The few twin studies that have been conducted on DMD have reported more consistent disease progression within twin pairs than amongst non-twin groups [16,17,18]. This, as would be expected, suggests that disease progression has a strong genetic component. Although sample sizes for GWAS studies has been limited, several genetic modifiers have now been identified.

In relation to physical activity, there are currently no reports in the literature that have directly examined the relationship of physical activity levels to disease progression in a cause and effect relationship. From what is known about variable responses to exercise, it is likely that activity levels play a role in the variability of disease progression. If higher levels of physical activity are related to slower disease progression, activity might be beneficial or those with slower disease progression might be more likely to be more active. Teasing apart the cause and effect will not be trivial, but a combination of associative human studies (of physical activity and disease progression), and animal intervention studies, will provide valuable insight into this process. In animals, voluntary exercise has been shown to be beneficial [19]. Methods such as using running wheel locks, when a voluntary exercise dose is reached, can be used to examine these questions directly. Currently, there is a need for these studies to determine how much variance is due to physical activity.

## 3. Genetic Modifiers

Genetic modifiers are typically discussed as genes or gene/protein expression patterns that can modify a disease or the progression of a disease. In DMD, animal models have yielded important insights into protein and protein pathways that can affect disease, and these can be very valuable in the design of new treatments, both as direct targets and in describing the disease pathology. However, these are unlikely to be used in the context of precision medicine unless there is a known variability that occurs naturally in these genes that can explain phenotype and guide treatment. The current focus here is, therefore, the genetic modifiers identified from the study of affected patient populations to identify common genomic variations. In precision medicine, these findings could provide the most useful results in terms of translation in the clinic. Ideally, the study in this context would utilize large databases of tens of thousands of individuals and use an unbiased genome-wide examination of related polymorphisms in comparison to the most important and consistent clinical outcomes of the disease. Studying rare genetic diseases is, unfortunately, inherently problematic for this optimal study design. Sample sizes tend be relatively small, and in DMD, important disease outcomes, such as loss of ambulation, are to some degree a combination of function and behavior and not always consistently defined across studies or cohorts. With these limitations in mind, genetic variations have still been identified.

Inflammation and fibrosis are the primary treatment targets, as evidenced through the optimization of corticosteroid treatments in the clinic and the continued research on the TGF-Beta (Transforming Growth Factor Beta) pathway in skeletal muscles. In this regard, the osteopontin gene has been considered a critical regulator of this pathway. Osteopontin is a secreted phosphoprotein (SPP1) that regulates immune, bone, and muscle physiology. A SNP that affects transcriptional activity was identified in the SPP1 promoter [20]. Subsequently, the SPP1 protein was shown to be elevated in DMD patient muscles and the serum of mdx mice [21], while mechanistic studies confirmed a role for SPP1 in promoting fibrosis in the mdx mouse [22]. The SPP1 SNP rs 28357094 was then examined in two separate cohorts of DMD patients (n = 106 & n = 156) [23]. The two-cohort design was used to both identify and validate polymorphisms related to DMD disease severity. The SPP1 SNP rs 28357094 was related to disease severity. At least one subsequent study was able to replicate these findings [24], while others did not [25,26]. These non-confirmatory studies did not find the SPP1 genotype as predictive alone of disease severity but did identify a TGF-BetaR2 SNP rs4522809 as being predictive of osteopontin expression and identified the haplotype in the LTBP4 gene as predictive of disease severity. Admittedly, small sample sizes and statistically insensitive measures, such as age at loss of ambulation, make it difficult to find small effects, yet these findings further implicate the TGFB inflammatory pathway as an important disease modifier. Also notable is the association found between the SPP1 SNP when steroid treatment is included in the analysis [24]. This suggests that the gene effect can be medication specific, further aiding idea of precision medicine. In regard to inflammation specifically, the rs1883832 SNP of CD40 T-cells was recently identified as a disease modifier [27], again implicating its inflammatory state and variations as important for disease progression. The only SNP identified that is not part of the inflammatory cascade is the ACTN3 (Alpha-actinin 3) genotype, which causes a loss of the protein in carriers of this mutation and was shown to affect DMD [28]. ACTN3 is a structural protein located at the Z-disc of muscle sarcomeres, where it anchors actin myofilaments. The (rs 1815739) polymorphism causes a premature stop codon and, therefore, allows no ACTN3 to be expressed in fast twitch skeletal muscle fibers. This polymorphism has been associated with muscle performance in healthy people as well as in people with DMD [29].

As more polymorphisms are identified and interactions between them are explored, it will be possible to use polymorphisms in precision treatments. Indeed, steroid treatment seems to be a co-determinant of the SPP1 SNP effect. Identifying other treatments that interact with genotype to predict disease outcome could greatly aid in personalizing treatment, by precisely selecting and optimizing treatment. Gene therapy treatments might interact, lessen, or strengthen some of these genotype associations. SNPs could be used to develop new taxonomies or subcategories of disease to better predict who will respond to certain therapies. In this regard, it is very likely that physical activity and/or therapeutic exercise will interact with the SNPs. Numerous SNPs that affect the exercise response in healthy and disease populations have been identified, as will be discussed in this paper. It is then possible that some boys with DMD or BMD may respond positively to increased physical activity, although there is, admittedly, little evidence to currently support this. It is likely that in the future, in combination with other therapies, boys with certain SNPs will respond better to certain gene therapies and certain exercise interventions. These combinations, producing a synergistic effect, are an ideal example of the promise of precision medicine.

## 4. Gene, Molecular, and Cellular Therapies

The idea of human gene modification to cure disease goes back at least 40 years [30]. In the 1990s, the development of adenoviral vectors (AVs) seemed to finally deliver on this promise. The further development of lentiviruses (LVs) added additional hope. Several clinical trials were run, with some ending in disastrous consequences [31,32]. The viral vectors were not completely understood and resulted in unexpected toxicities. Several years of basic bench science on the biology of these viral delivery vectors, and their interaction with the human immune system, resulted in major advancements that have led to renewed interest and successes in virally delivered genetic material. Part of this success has been the development of viruses, including adeno-associated viruses (AAV). Additionally, the development of chemistries that can induce designed and specific alternative splicing in human cells offers the opportunity to “skip” or splice over mutations to create truncated proteins; in many cases, this process is enough to offer at least a partial functional protein correction in the cell. Finally, the advancement in genome editing techniques, including the recent discovery of CRISPR-Cas9, has allowed the potential to literally correct the disease-causing mutations in the cell. Some other advanced biologic therapies that are demonstrating great promise include modifying the expression of Utrophin or other structural muscle genes, and cellular therapies. All of these therapies are in some stage of clinical trial or clinical trial development, but it does not currently appear that there will be one best answer for all boys with DMD.

The viral vector delivery of genetic material was the first attempt at gene therapy. The overall idea is simple, albeit difficult in reality to safely achieve. Genetic material that encodes the correct sequence of a gene that has a disease-causing mutation is encapsidated into a virus, which is then delivered to an organ directly or systemically, with the intention that the virus will bind to target cells and deliver the genetic material to those affected cells for gene expression and possible incorporation into the genome. There are advantages and disadvantages to AAVs and LVs that are beyond the scope of this review, but a few are unique to muscle and dystrophin. AAVs tend to offer longer term transgene expression in non-dividing cells, such as muscle fibers, but dividing fibers are better infected by LVs. Lentiviruses integrate into the genome, conferring infected cells the ability to transfer the transgene to daughter cells, a key advantage for long term therapy and for organs with a regenerative capacity, like skeletal muscle. Additionally, LV’s can carry up to 10 kb of genetic material while the newest AAV’s have an encapsidation ability of up to ~5 kb [33]. Considering that the transcript length of dystrophin is 14 kb, this storage capacity is an important difference and has led to research in truncating the gene before delivery.

Improvements in our understanding of the essential components of the dystrophin protein have yielded mini and microdystrohin genes used with these small encapsidation capacity viral vectors. Indeed, several clinical trials have been undertaken. However, because of these and other limitations, it does not appear that any of the current viral dystrophin delivery methods will be able to infect 100% of the muscle fibers. A cautiously optimistic estimate might be a 60%–70% fiber transfection after several more years of research, and this amount will likely require multiple treatments or at least periodic boosters throughout a lifetime. Thus, a Becker-like phenotype will be created, which could extend a lifespan to at least the 60s or 70s. These patients will certainly become more active and some may desire to become very active. We do not know how exercise will affect patients after the therapy, or how exercise might interact with, or improve, the therapy itself. Exercise has significant effects on the immune system and inflammation acutely (in response to one exercise session) and chronically (in response to long-term training). Additionally, some forms of muscle contraction (e.g., eccentric contractions) cause muscle membrane micro-tears, leakage, and repair, and have acute and chronic effects on satellite cell division. Through this opening and resealing of the muscle membrane, exercise is, therefore, very likely to affect viral uptake into dividing and non-dividing cells, in addition to signaling inflammation, growth, and repair pathways, and will have a substantial long-term impact on this type of therapy.

Another very promising approach related to gene delivery is exon skipping. Several different classes of drugs and chemical compounds have now been shown to alter mRNA splicing, to skip over the mutated exon. This therapeutic approach would require lifetime treatment, but would also avoid some of the major complications of the viral delivery methods. Yet, exon skipping strategies will not be able to treat all boys. Currently, the drugs in development target the most common mutations that cause DMD and can, in theory, currently treat around 13% of all boys. With continued development, it is estimated that exon skipping therapies could be used to treat up to about 80% of all boys [34]. These treatments, once optimized, will again be unlikely to treat 100% of the cells and in the cells that are affected it is unlikely that dystrophin would reach 100%. Once again, this treatment creates a milder, Becker-like pathology. The effect of exercise here is unknown. Exercise could modify the uptake of the drug or could separately affect its ability to work, as exercise is known to affect gene and protein expression of dystrophin, utrophin, and other structural proteins [35]. Exercise also effects numerous signaling pathways in muscles, some of which are directly related to, or control, gene splicing [36]. It seems very likely that exercise of the correct dose would increase the ability of the drug to work, whether or not it affected drug uptake. Direct experiments will be needed.

### 4.1. Genome Editing

To be able to physically correct the DNA mutation causing the disease would be the ultimate goal of a gene-type therapy for DMD. To directly target the DNA of each cell, remove the mutation, and replace it with the correct sequence would, in theory, be considered a cure for this genetic disease. The advent of CRISPR/Cas9 technology now opens this possibility. Optimization is needed, including delivery of the protein machinery, precise control of DNA correction with no off-target effects, and the ability to turn this machinery off when it is no longer needed. Other methods, including zinc-finger nucleases and transcription activator-like effector nucleases (TALENs), are targeting nucleases that still hold potential [37]. However, delivery of these gene correcting proteins remains an obstacle for human patients. Even when delivery (likely by a viral vector) is optimized, off-target mutation is still a concern [38,39]. There is also the possibility of combining this therapy with cell-based therapy, by removing cells from a patient, modifying them outside the body, and delivering them back into the tissues of interest. This delivery could be systemic or localized. This process has been successfully accomplished with mdx mice [40], yet being able to correct enough cells, and getting enough of them to engraft correctly into the muscle tissue of humans remains a major obstacle. Because it would be unlikely to treat all cells, this process would also create a new Becker-like phenotype. Because of the variety of delivery methods that could be used for genome editing, it remais difficult to anticipate what the interactions would be with exercise. Factors such as inflammation, satellite cell activation and localization, and endogenously activated tissue repair pathways are all affected by exercise and could interfere with or enhance the response to these treatments. The stage of the disease is also likely to interact with treatment and exercise response. The length of time the patient had the disease before it was fully treated would affect the exercise response, as the patient would have at least some, or perhaps extensive, fibrosis before the remaining muscle cells were corrected. How exercise therapy could be used to improve muscle function in these patients is something that will require dedicated and specific research.

### 4.2. Cell-Based Therapies

The goal of cell-based therapies is to deliver cells that possess an intact dystrophin gene and integration of these cells with the muscle tissue. Ideally, at least some of these cells could act as satellite cells that could divide and replenish their own satellite cell niche while allowing new cells to functionally integrate with the tissue. Cells could be delivered systemically or directly into the tissue, and while these methods have proved somewhat successful in animal experiments [41], human experiments have failed to produce good results [42,43]. The number of cells needed, getting them to cross the epithelial layer and to remain in the muscle, and being able to replace the satellite cell niche are some of the primary obstacles. Another obstacle is the source of the cells. As mentioned CRISPR could use the patient’s own cells, embryonic stem cells, or pluripotent stem cells. Additionally, some rarer cell types, such as CD133+, have been considered, but not much work has been done in this area [44]. However, at best this would likely be an adjuvant therapy or only produce a Becker-like phenotype. The exercise response would, perhaps, be the most difficult to predict of all the therapies mentioned, due to the different types of cellular therapy options that could be available. However, exercise would certainly effect the cells’ ability to find and integrate at the correct location, and organize correctly across lines of stress induced by exercise. Exercise would impact local and systemic production of growth factors and would be critical for correct nervous system innervation.

Because success of these therapies will be at least partially dependent upon the immune system, and the known and unknown ways that exercise interacts with the immune system, it seems imperative that, as new therapies and drugs are developed, in the early stages of development, exercise related experiments are conducted to understand how exercise interacts with these treatments to increase or decrease their effectiveness, and also to examine more precise exercise prescriptions. Another factor not yet mentioned is how these therapies affect heart tissue. It is the hope that many of the therapies will positively impact cardiac tissue. Certainly, exercise has numerous acute and chronic effects on the heart, including mechanical, hormonal, and neural. The treatment of cardiac tissue in DMD has, furthermore, been an obstacle, and exercise may be able to enhance the efficacy of new treatments. As mentioned, these improved treatments will eventually lead to the creation of a Becker-like phenotype. Knowing how different types of exercise will then affect these new Becker-like phenotypes will be essential for a real-world optimized treatment.

## 5. Biomarkers

As advanced therapies are examined and improved, the number of clinical trials will continue to increase. However, precisely monitoring these therapies is not trivial for a progressive and variable disease. Therapies will be delivered at different disease stages, and what is a clinical marker of disease worsening versus disease improvement could vary with disease stage. Identifying clinical markers that can be used with any new treatment will be necessary to track disease progression and is a cornerstone of personalized medicine. DMD, being a rare genetic disease, makes this process especially difficult. Samples sizes in a DMD clinical trial will never reach the sample size of cardiovascular disease or diabetes studies, which can easily be in the thousands. Currently, the two best outcome measures when examining disease progression or treatment effectiveness are dystrophin protein expression and the six minute walk test. Dystrophin protein expression requires a muscle biopsy, often of a substantial size, if immunohistochemistry is to be conducted. This procedure is invasive and painful for the patient and somewhat expensive, technical, and time consuming for the analysis. The biopsy is only a small piece of just one muscle, which is not necessarily reflective of the entire musculature. The six minute walk test, which is the most commonly used functional test, is highly variable, dependent on patient motivation, fatigue, coaching, can only be used in ambulant patients, and increases the risk of falls. Thus, there is a critical need for biomarkers that are acute sensitive measures of treatment effects and predictive of benefit or outcomes.

The two most promising types of biomarkers for DMD appear to be miRNAs or proteins from blood samples. Progress has been made in both of these biomarkers, but as the disease progresses, the biomarkers patterns change. Thus, a biomarker of a young boy with the disease who still has substantial muscle mass and is somewhat active is likely to have a very different biomarker profile than a patient in his 20s who has lost much of his muscle tissue and is very inactive. This will have to be considered when formalizing disease biomarkers. Identifying one biomarker will be extremely unlikely, but using several to establish a pattern is most likely to work. A recent review highlighted this idea by classifying discovered biomarkers into categories that appeared to relate to disease progression and age [45].

The most consistently used diagnostic marker for DMD has been muscle specific creatine kinase (CK). As muscle cells are damaged and/or become necrotic, CK protein leaks from its intracellular location to the blood. CK is, however, highly variable between patients, even those of a similar age, and blood values are susceptible to physical activity, exercise, and amount of muscle mass. CK is, therefore, not thought to be a reliable and precise maker of disease severity. A potential breakthrough in this area came in the form of miRNAs. One of the first DMD related studies to report miRNAs in blood samples identified miR-1, miR-20, and miR-133 as being able to differentiate between DMD patients and healthy controls and also between the less severe form of DMD, Becker Muscular Dystrophy (BMD) [46]. The miRNAs were also elevated in the mdx mouse model of DMD and were responsive to dystrophin replacing treatment. Being able to diagnose, differentiate severity, and respond to treatment are all essential criteria for use as a biomarker. Other animal models beyond the mdx mouse have further supported these findings [47].

However, complications remain, as these miRNAs may still suffer from similar variations to CK, because they often leak from damaged fibers, remain age dependent [46,48], and exercise and muscle repair can increase miRNA in both healthy controls and in the mdx mouse [48,49,50]. The magnitude of change in miRNA is less than that of CK in response to exercise, which points to the miRNA being a more stable measure. However, an increase in miRNA is related to muscle repair and damage [51,52]. In this regard, it is interesting that while circulating miRNAs increase, this circulation is not necessarily reflective of any tissue changes, and some miRNAs of the greatest abundance in muscle tissue are not changed in the circulation upon damage. This phenomenon points to a selective release of miRNA from muscle as opposed to just representing damaged tissue. If the release were selective, consistent, and related to disease progression, it would be a vast improvement over the non-specific CK release. Furthermore, selectively released miRNAs are known to have effects on neighboring or distant tissues in the body, which might further indicate specific disease progression. In a recent comprehensive review of miRNAs in dystrophy, the authors suggest four processes as representative of circulating miRNAs: (1) cell release due to myonecrosis, (2) passive leakage due to membrane damage, (3) abnormal secretion due to dystrophin deficiency, and (4) selective release related to muscle repair and regeneration. Processes 2 and 4 would be affected by exercise interventions and should be considered in these studies. Studies that directly compare exercise interventions of healthy animals to dystrophic animals would help elucidate biomarkers indicative of a healthy muscle repair process, as opposed to those with an inability to repair muscle, such as those in DMD.

In addition to miRNAs, proteins are also being used as biomarkers. Many muscle specific proteins will leak from the damaged muscle cells, similar to CK. The goal is to identify those biomarkers that are specific to DMD and are accurate/precise in determining the effect of therapeutic interventions. Two primary discovery methods for protein-based biomarkers are to use affinity-based arrays for known proteins and to use the less biased proteomics, utilizing mass spectrometry. Each method has benefits and drawbacks, and both have produced proteins that could be used to monitor DMD. There markers still suffer from some of the same problems as CK, because they are dependent upon age, muscle mass, or the state of the disease, but when these variables are accounted for, these different markers could be used during different stages of the disease or to monitor various part of the pathophysiology. In a recent review focused on proteomic approaches in DMD, the authors classified newly-discovered proteins, based on their pathobiochemical pathway, into four categories. These are (1) cell membrane leakage, (2) Fibrosis, (3) Inflammation, and (4) muscle degeneration and regeneration [45]. Additional studies could help to further classify these disease categories, determine at what stage of the disease each might be the most important, and monitor each in response to a treatment. Because exercise is likely to have significant impacts on categories 2, 3, and 4, studies need to compare the response of exercise in healthy people to these categories in DMD patients. Exercise can be used as a valuable model to identify the biomarkers of healthy muscle damage, inflammation, and repair compared to the failure to regenerate seen in DMD. In the short term, studies in the mouse models could move this field forward substantially, where variability in many measures—functional, physiological, cellular and molecular—are quantified in relation to each of these biomarker categories, in response to exercise interventions. This would develop panels of biomarkers that represent healthy muscle repair for several functional, physiologic, and molecular outcomes. There is a dearth of data in this area and a need for research. It does seem certain that a panel of biomarkers will be needed to categorize, decide upon, and monitor treatments for precision medicine, and exercise interventions should be part of this equation.

## 6. Physiologic Variability Inherent in Exercise Training

While physical activity is recommended by the surgeon general of the United States and essentially every major medical organization as both a preventative measure, and sometimes a treatment, for many of the most common chronic diseases in the developed world today (CVD, obesity, diabetes, etc.) it is also recognized that there can be an extreme variability between individual responses to exercise training. For instance, in response to exercise training, changes in variables, such as maximal oxygen consumption (VO2max) can vary greatly (e.g., 0–100%) [53,54], and changes in response to resistance training, such as maximal strength, vary even more (e.g., 0–250%) [55]. This variability has been extended to measures of muscle fiber size, as well [56]. For metabolic factors, exercise is a well-characterized treatment for diabetes, and while some patients who begin a program substantially improve many measures of metabolism, others experience an adverse metabolic response to exercise [57]. Certainly, some studies have found that if an exercise dose is increased, even the non-responders experience benefits. These studies showed that it is more a question of a more individualized exercise dose than a person not responding to exercise [58]. Nevertheless, some people, even amongst generally healthy individuals, do not derive much benefit from a particular exercise intervention for a particular outcome variable. Considering, then, the variability inherent in a neuromuscular disease such as DMD, as mentioned earlier in this paper, it is expected that the exercise response would be even more variable in this population. There has been very little research in this area where a response or non-response could be determined based on groups. Understandably, there is a paucity of research in this area, as intense physical exercise can cause further harm to patients with DMD. Responsive and non-responsive outcome groups of exercise studies with animals could produce valuable insight. In some individuals, there could be a tremendously positive impact that is nonexistent when a group average is taken. Currently, we have no way of categorizing or classifying those that might respond.

## 7. The Future of Precision Exercise Therapy in DMD

A recent review thoroughly examined the animal and human literature regarding exercise interventions in DMD [19]. To summarize the animal studies, they suggest that high-intensity, high-volume, eccentric muscle contractions exacerbate the disease pathology, but that lower intensity and volume of exercise are unlikely to exacerbate disease pathology and actually seem to be beneficial. Thus, low intensity and low impact exercises, such as bicycle training, body weight-supported walking, and aquatic exercise, should be beneficial in most circumstances. The largest gap in the animal literature is in our understanding of the long-term exercise effects on the cardiopulmonary system. However, a report published after the aforementioned review examined exercise effects in mice with no dystrophin and mice with very low levels of dystrophin and found no increase in pathology and improvements in skeletal muscle and cardiac function [59]. The mice with low levels of dystrophin improved more than those without dystrophin. This data supports the idea of precision medicine for exercise prescription. Future studies that examine exercise dose and combination treatments are still needed.

There are very few human studies that have examined exercise in DMD and BMD patients. In general, these studies conclude that low intensity exercise does not appear to damage the muscle and is likely beneficial [60,61,62,63,64,65]. Caution is certainly warranted here, with so few studies and very small sample sizes, but it does appear that self-limited low intensity/volume exercise is beneficial to at least some patients with DMD and BMD. More long-term studies are needed, especially those that examine the effects on the cardiopulmonary system.

In conclusion, there is currently a need for exercise to be incorporated into most current therapies and biomarkers being developed for DMD, and a need for independent exercise physiology research on DMD, to better understand the effects of increased muscular activity on muscle that is affected by DMD. For patients with DMD, physical therapy and occupational therapy are very often standard care, while PT is likely to focus on splinting, to prevent contractures and maintain flexibility, and non-weight bearing physical activity, such as water exercises, to provide a mild muscle stimulation. Physical therapists have a vast anatomical and physiologic knowledge of responses to physical interventions and many therapeutic modalities at their disposal, but very few of these features are utilized in assisting boys affected by DMD, their families, and their caregivers. A form of precision exercise therapy could be envisioned, where physical activity (monitored with an activity monitor), diet, exercise, and physical therapy interventions would be compared to a panel of biomarkers and a physical outcome (strength measure) to optimize exercise and PT interventions for boys with DMD. A specialized PT could use this data in a multifactorial analysis to decide on exercise interventions or therapeutic modalities that would optimize an exercise intervention. Genetics and innovative treatments, along with the most recent measures of muscle function or MRI of muscle to monitor damage, would be considered. The PT could work directly with the neurologist, radiologist (for muscle imaging), boy, and his family to decide on the best exercise interventions to maintain or improve muscle function and quality of life.
